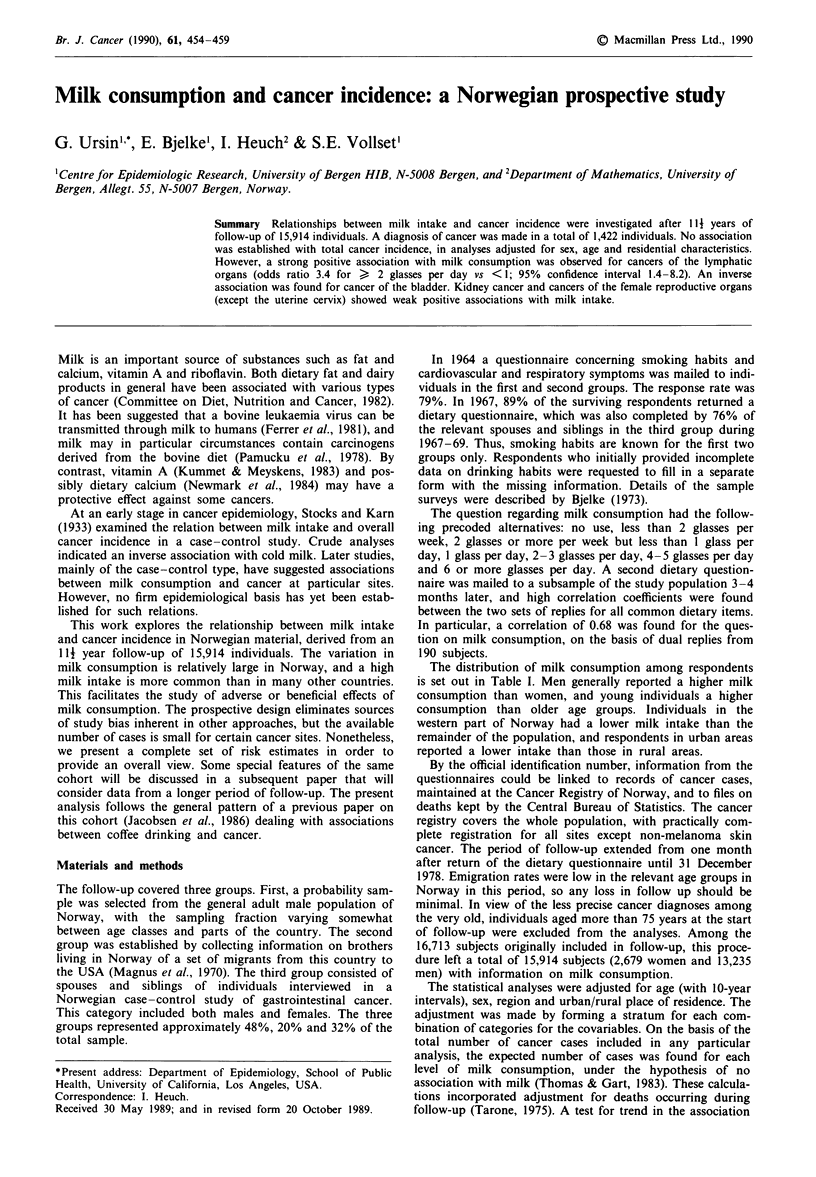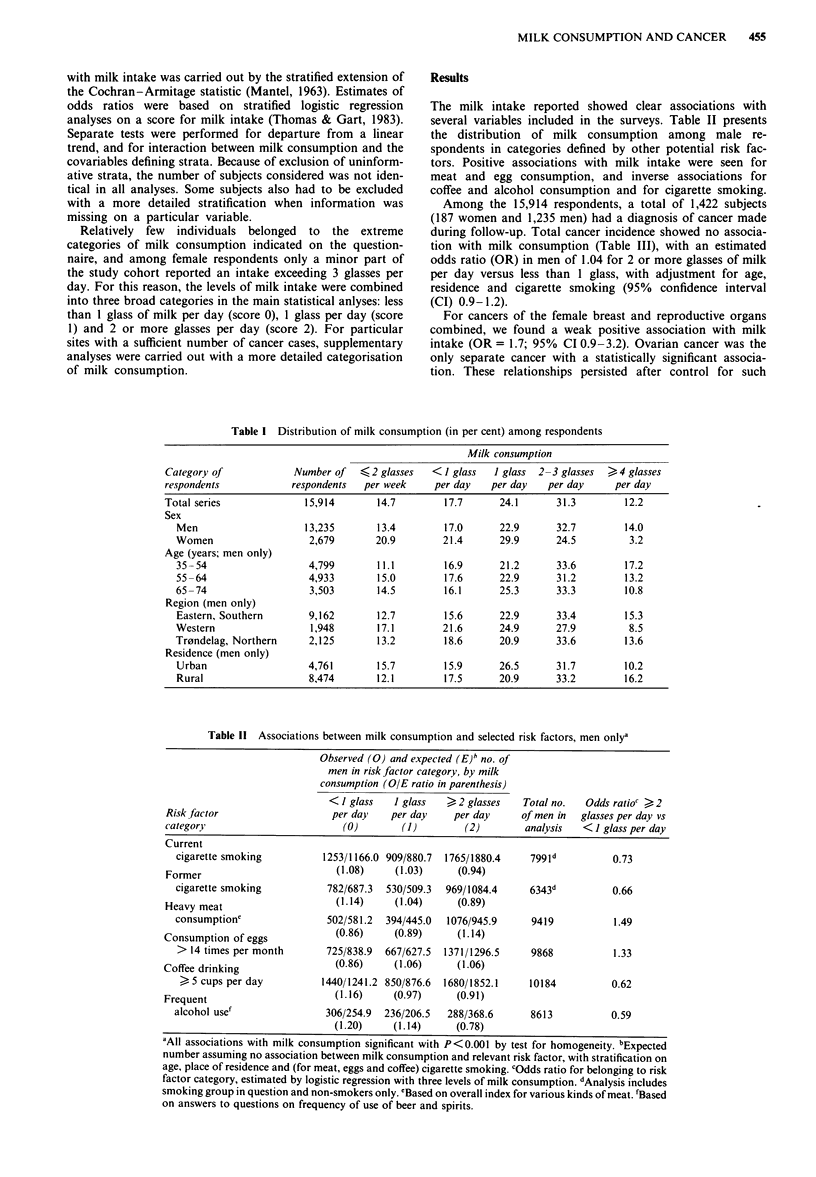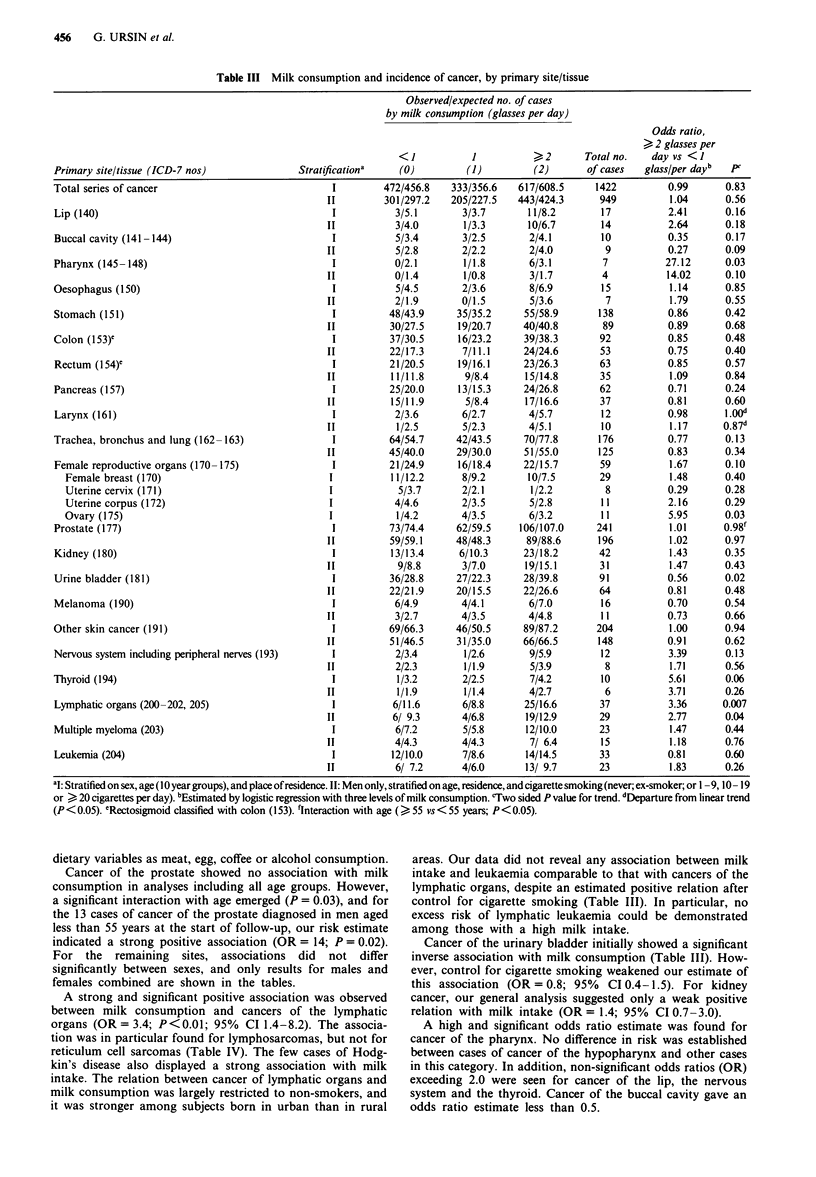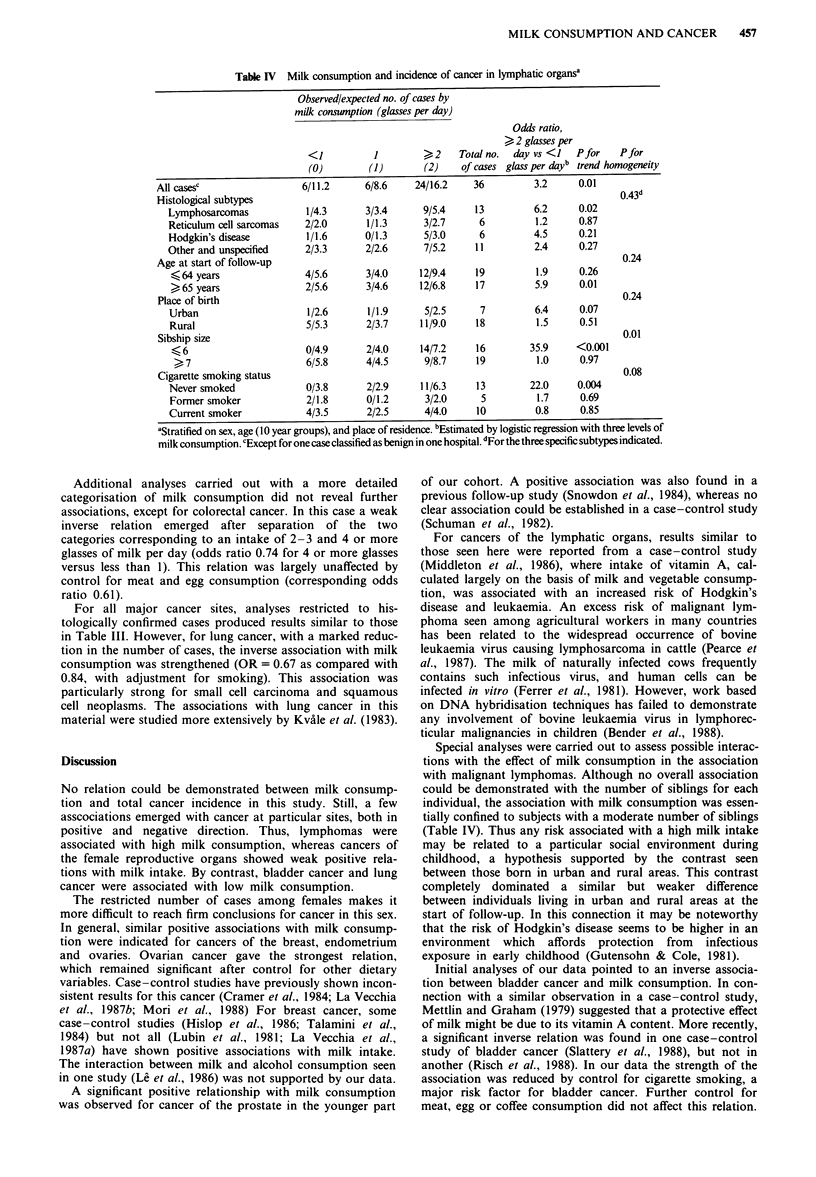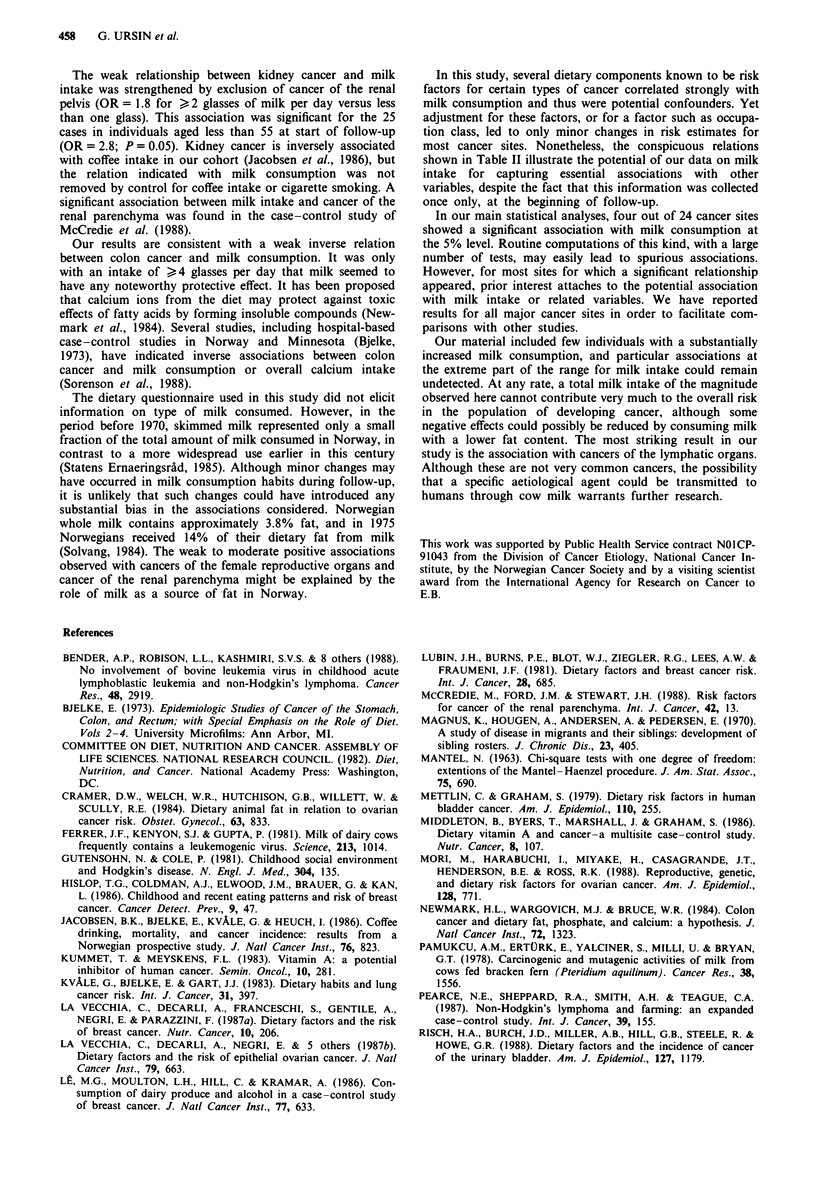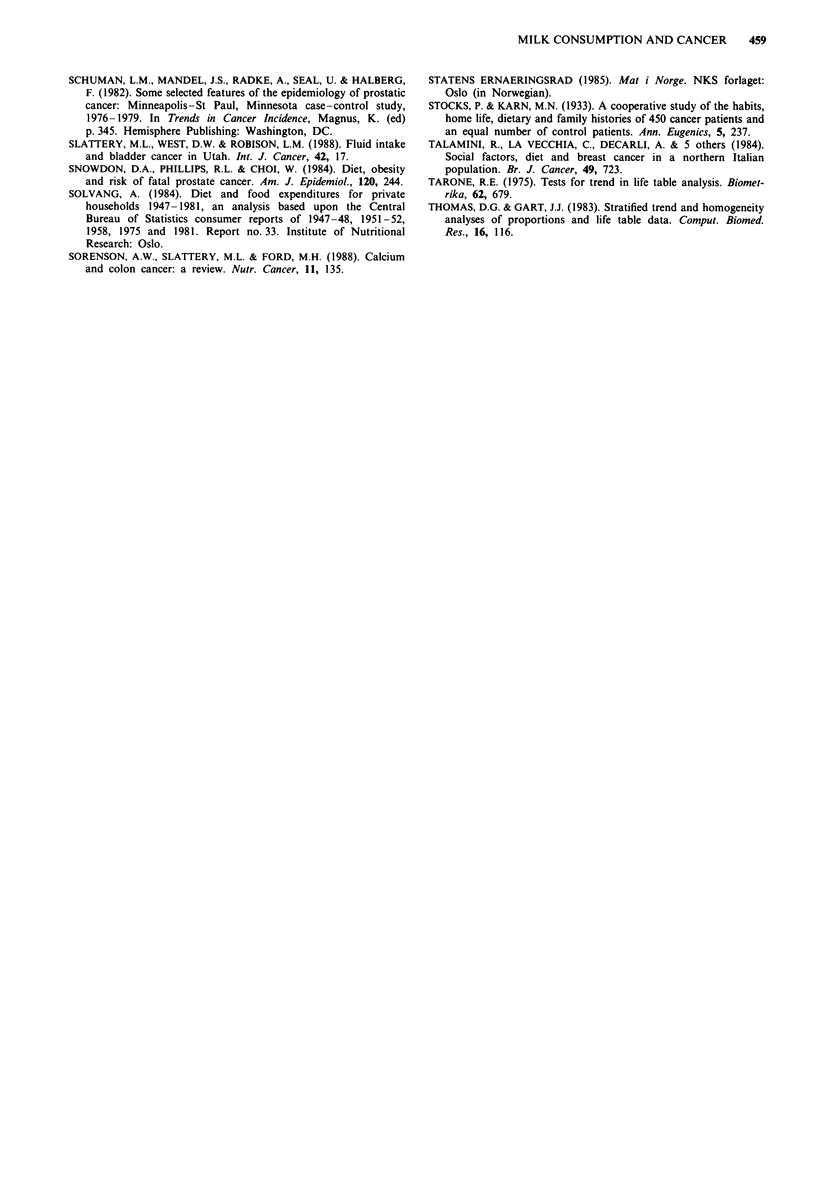# Milk consumption and cancer incidence: a Norwegian prospective study.

**DOI:** 10.1038/bjc.1990.100

**Published:** 1990-03

**Authors:** G. Ursin, E. Bjelke, I. Heuch, S. E. Vollset

**Affiliations:** Centre for Epidemiologic Research, University of Bergen HIB, Norway.

## Abstract

Relationships between milk intake and cancer incidence were investigated after 11 1/2 years of follow-up of 15,914 individuals. A diagnosis of cancer was made in a total of 1,422 individuals. No association was established with total cancer incidence, in analyses adjusted for sex, age and residential characteristics. However, a strong positive association with milk consumption was observed for cancers of the lymphatic organs (odds ratio 3.4 for greater than or equal to 2 glasses per day vs less than 1; 95% confidence interval 1.4-8.2). An inverse association was found for cancer of the bladder. Kidney cancer and cancers of the female reproductive organs (except the uterine cervix) showed weak positive associations with milk intake.


Br. J. Cancer (1990), 61, 454-459 © Macmillan Press Ltd., 1990

### Milk consumption and cancer incidence: a Norwegian prospective study

G. Ursin[1,*], E. Bjelke[1], I. Heuch[2] & S.E. Vollset[1]

[1]*Centre for Epidemiologic Research, University of Bergen HIB, N-5008 Bergen, and* [2]*Department of Mathematics, University of Bergen, Allegt. 55, N-5007 Bergen, Norway.*

**Summary** Relationships between milk intake and cancer incidence were investigated after 11½ years of follow-up of 15,914 individuals. A diagnosis of cancer was made in a total of 1,422 individuals. No association was established with total cancer incidence, in analyses adjusted for sex, age and residential characteristics. However, a strong positive association with milk consumption was observed for cancers of the lymphatic organs (odds ratio 3.4 for $\geqslant$ 2 glasses per day *vs* $<1$; 95% confidence interval 1.4-8.2). An inverse association was found for cancer of the bladder. Kidney cancer and cancers of the female reproductive organs (except the uterine cervix) showed weak positive associations with milk intake.

Milk is an important source of substances such as fat and calcium, vitamin A and riboflavin. Both dietary fat and dairy products in general have been associated with various types of cancer (Committee on Diet, Nutrition and Cancer, 1982). It has been suggested that a bovine leukaemia virus can be transmitted through milk to humans (Ferrer *et al.*, 1981), and milk may in particular circumstances contain carcinogens derived from the bovine diet (Pamucku *et al.*, 1978). By contrast, vitamin A (Kummet & Meyskens, 1983) and possibly dietary calcium (Newmark *et al.*, 1984) may have a protective effect against some cancers.

At an early stage in cancer epidemiology, Stocks and Karn (1933) examined the relation between milk intake and overall cancer incidence in a case–control study. Crude analyses indicated an inverse association with cold milk. Later studies, mainly of the case–control type, have suggested associations between milk consumption and cancer at particular sites. However, no firm epidemiological basis has yet been established for such relations.

This work explores the relationship between milk intake and cancer incidence in Norwegian material, derived from an 11½ year follow-up of 15,914 individuals. The variation in milk consumption is relatively large in Norway, and a high milk intake is more common than in many other countries. This facilitates the study of adverse or beneficial effects of milk consumption. The prospective design eliminates sources of study bias inherent in other approaches, but the available number of cases is small for certain cancer sites. Nonetheless, we present a complete set of risk estimates in order to provide an overall view. Some special features of the same cohort will be discussed in a subsequent paper that will consider data from a longer period of follow-up. The present analysis follows the general pattern of a previous paper on this cohort (Jacobsen *et al.*, 1986) dealing with associations between coffee drinking and cancer.

### Materials and methods

The follow-up covered three groups. First, a probability sample was selected from the general adult male population of Norway, with the sampling fraction varying somewhat between age classes and parts of the country. The second group was established by collecting information on brothers living in Norway of a set of migrants from this country to the USA (Magnus *et al.*, 1970). The third group consisted of spouses and siblings of individuals interviewed in a Norwegian case–control study of gastrointestinal cancer. This category included both males and females. The three groups represented approximately 48%, 20% and 32% of the total sample.

In 1964 a questionnaire concerning smoking habits and cardiovascular and respiratory symptoms was mailed to individuals in the first and second groups. The response rate was 79%. In 1967, 89% of the surviving respondents returned a dietary questionnaire, which was also completed by 76% of the relevant spouses and siblings in the third group during 1967–69. Thus, smoking habits are known for the first two groups only. Respondents who initially provided incomplete data on drinking habits were requested to fill in a separate form with the missing information. Details of the sample surveys were described by Bjelke (1973).

The question regarding milk consumption had the following precoded alternatives: no use, less than 2 glasses per week, 2 glasses or more per week but less than 1 glass per day, 1 glass per day, 2–3 glasses per day, 4–5 glasses per day and 6 or more glasses per day. A second dietary questionnaire was mailed to a subsample of the study population 3–4 months later, and high correlation coefficients were found between the two sets of replies for all common dietary items. In particular, a correlation of 0.68 was found for the question on milk consumption, on the basis of dual replies from 190 subjects.

The distribution of milk consumption among respondents is set out in Table I. Men generally reported a higher milk consumption than women, and young individuals a higher consumption than older age groups. Individuals in the western part of Norway had a lower milk intake than the remainder of the population, and respondents in urban areas reported a lower intake than those in rural areas.

By the official identification number, information from the questionnaires could be linked to records of cancer cases, maintained at the Cancer Registry of Norway, and to files on deaths kept by the Central Bureau of Statistics. The cancer registry covers the whole population, with practically complete registration for all sites except non-melanoma skin cancer. The period of follow-up extended from one month after return of the dietary questionnaire until 31 December 1978. Emigration rates were low in the relevant age groups in Norway in this period, so any loss in follow up should be minimal. In view of the less precise cancer diagnoses among the very old, individuals aged more than 75 years at the start of follow-up were excluded from the analyses. Among the 16,713 subjects originally included in follow-up, this procedure left a total of 15,914 subjects (2,679 women and 13,235 men) with information on milk consumption.

The statistical analyses were adjusted for age (with 10-year intervals), sex, region and urban/rural place of residence. The adjustment was made by forming a stratum for each combination of categories for the covariables. On the basis of the total number of cancer cases included in any particular analysis, the expected number of cases was found for each level of milk consumption, under the hypothesis of no association with milk (Thomas & Gart, 1983). These calculations incorporated adjustment for deaths occurring during follow-up (Tarone, 1975). A test for trend in the association

*Present address: Department of Epidemiology, School of Public Health, University of California, Los Angeles, USA.
Correspondence: I. Heuch.
Received 30 May 1989; and in revised form 20 October 1989.

with milk intake was carried out by the stratified extension of the Cochran–Armitage statistic (Mantel, 1963). Estimates of odds ratios were based on stratified logistic regression analyses on a score for milk intake (Thomas & Gart, 1983). Separate tests were performed for departure from a linear trend, and for interaction between milk consumption and the covariables defining strata. Because of exclusion of uninformative strata, the number of subjects considered was not identical in all analyses. Some subjects also had to be excluded with a more detailed stratification when information was missing on a particular variable.

Relatively few individuals belonged to the extreme categories of milk consumption indicated on the questionnaire, and among female respondents only a minor part of the study cohort reported an intake exceeding 3 glasses per day. For this reason, the levels of milk intake were combined into three broad categories in the main statistical anlyses: less than 1 glass of milk per day (score 0), 1 glass per day (score 1) and 2 or more glasses per day (score 2). For particular sites with a sufficient number of cancer cases, supplementary analyses were carried out with a more detailed categorisation of milk consumption.

### Results

The milk intake reported showed clear associations with several variables included in the surveys. Table II presents the distribution of milk consumption among male respondents in categories defined by other potential risk factors. Positive associations with milk intake were seen for meat and egg consumption, and inverse associations for coffee and alcohol consumption and for cigarette smoking.

Among the 15,914 respondents, a total of 1,422 subjects (187 women and 1,235 men) had a diagnosis of cancer made during follow-up. Total cancer incidence showed no association with milk consumption (Table III), with an estimated odds ratio (OR) in men of 1.04 for 2 or more glasses of milk per day versus less than 1 glass, with adjustment for age, residence and cigarette smoking (95% confidence interval (CI) 0.9–1.2).

For cancers of the female breast and reproductive organs combined, we found a weak positive association with milk intake (OR = 1.7; 95% CI 0.9–3.2). Ovarian cancer was the only separate cancer with a statistically significant association. These relationships persisted after control for such

**Table I** Distribution of milk consumption (in per cent) among respondents

| Category of respondents | Number of respondents | Milk consumption: ⩽2 glasses per week | < 1 glass per day | 1 glass per day | 2–3 glasses per day | ⩾4 glasses per day |
|---|---|---|---|---|---|---|
| Total series | 15,914 | 14.7 | 17.7 | 24.1 | 31.3 | 12.2 |
| Sex | | | | | | |
| Men | 13,235 | 13.4 | 17.0 | 22.9 | 32.7 | 14.0 |
| Women | 2,679 | 20.9 | 21.4 | 29.9 | 24.5 | 3.2 |
| Age (years; men only) | | | | | | |
| 35–54 | 4,799 | 11.1 | 16.9 | 21.2 | 33.6 | 17.2 |
| 55–64 | 4,933 | 15.0 | 17.6 | 22.9 | 31.2 | 13.2 |
| 65–74 | 3,503 | 14.5 | 16.1 | 25.3 | 33.3 | 10.8 |
| Region (men only) | | | | | | |
| Eastern, Southern | 9,162 | 12.7 | 15.6 | 22.9 | 33.4 | 15.3 |
| Western | 1,948 | 17.1 | 21.6 | 24.9 | 27.9 | 8.5 |
| Trøndelag, Northern | 2,125 | 13.2 | 18.6 | 20.9 | 33.6 | 13.6 |
| Residence (men only) | | | | | | |
| Urban | 4,761 | 15.7 | 15.9 | 26.5 | 31.7 | 10.2 |
| Rural | 8,474 | 12.1 | 17.5 | 20.9 | 33.2 | 16.2 |

**Table II** Associations between milk consumption and selected risk factors, men only[a]

| Risk factor category | Observed (O) and expected (E)[b] no. of men in risk factor category, by milk consumption (O/E ratio in parenthesis): < 1 glass per day (0) | 1 glass per day (1) | ⩾2 glasses per day (2) | Total no. of men in analysis | Odds ratio[c] ⩾2 glasses per day vs < 1 glass per day |
|---|---|---|---|---|---|
| Current cigarette smoking | 1253/1166.0 (1.08) | 909/880.7 (1.03) | 1765/1880.4 (0.94) | 7991[d] | 0.73 |
| Former cigarette smoking | 782/687.3 (1.14) | 530/509.3 (1.04) | 969/1084.4 (0.89) | 6343[d] | 0.66 |
| Heavy meat consumption[e] | 502/581.2 (0.86) | 394/445.0 (0.89) | 1076/945.9 (1.14) | 9419 | 1.49 |
| Consumption of eggs > 14 times per month | 725/838.9 (0.86) | 667/627.5 (1.06) | 1371/1296.5 (1.06) | 9868 | 1.33 |
| Coffee drinking ⩾5 cups per day | 1440/1241.2 (1.16) | 850/876.6 (0.97) | 1680/1852.1 (0.91) | 10184 | 0.62 |
| Frequent alcohol use[f] | 306/254.9 (1.20) | 236/206.5 (1.14) | 288/368.6 (0.78) | 8613 | 0.59 |

[a]All associations with milk consumption significant with $P < 0.001$ by test for homogeneity. [b]Expected number assuming no association between milk consumption and relevant risk factor, with stratification on age, place of residence and (for meat, eggs and coffee) cigarette smoking. [c]Odds ratio for belonging to risk factor category, estimated by logistic regression with three levels of milk consumption. [d]Analysis includes smoking group in question and non-smokers only. [e]Based on overall index for various kinds of meat. [f]Based on answers to questions on frequency of use of beer and spirits.

**Table III** Milk consumption and incidence of cancer, by primary site/tissue

| Primary site/tissue (ICD-7 nos) | Stratification[a] | Observed/expected no. of cases by milk consumption (glasses per day) <1 (0) | 1 (1) | ≥2 (2) | Total no. of cases | Odds ratio, ≥2 glasses per day vs <1 glass/per day[b] | P[c] |
|---|---|---|---|---|---|---|---|
| Total series of cancer | I | 472/456.8 | 333/356.6 | 617/608.5 | 1422 | 0.99 | 0.83 |
| | II | 301/297.2 | 205/227.5 | 443/424.3 | 949 | 1.04 | 0.56 |
| Lip (140) | I | 3/5.1 | 3/3.7 | 11/8.2 | 17 | 2.41 | 0.16 |
| | II | 3/4.0 | 1/3.3 | 10/6.7 | 14 | 2.64 | 0.18 |
| Buccal cavity (141–144) | I | 5/3.4 | 3/2.5 | 2/4.1 | 10 | 0.35 | 0.17 |
| | II | 5/2.8 | 2/2.2 | 2/4.0 | 9 | 0.27 | 0.09 |
| Pharynx (145–148) | I | 0/2.1 | 1/1.8 | 6/3.1 | 7 | 27.12 | 0.03 |
| | II | 0/1.4 | 1/0.8 | 3/1.7 | 4 | 14.02 | 0.10 |
| Oesophagus (150) | I | 5/4.5 | 2/3.6 | 8/6.9 | 15 | 1.14 | 0.85 |
| | II | 2/1.9 | 0/1.5 | 5/3.6 | 7 | 1.79 | 0.55 |
| Stomach (151) | I | 48/43.9 | 35/35.2 | 55/58.9 | 138 | 0.86 | 0.42 |
| | II | 30/27.5 | 19/20.7 | 40/40.8 | 89 | 0.89 | 0.68 |
| Colon (153)[e] | I | 37/30.5 | 16/23.2 | 39/38.3 | 92 | 0.85 | 0.48 |
| | II | 22/17.3 | 7/11.1 | 24/24.6 | 53 | 0.75 | 0.40 |
| Rectum (154)[e] | I | 21/20.5 | 19/16.1 | 23/26.3 | 63 | 0.85 | 0.57 |
| | II | 11/11.8 | 9/8.4 | 15/14.8 | 35 | 1.09 | 0.84 |
| Pancreas (157) | I | 25/20.0 | 13/15.3 | 24/26.8 | 62 | 0.71 | 0.24 |
| | II | 15/11.9 | 5/8.4 | 17/16.6 | 37 | 0.81 | 0.60 |
| Larynx (161) | I | 2/3.6 | 6/2.7 | 4/5.7 | 12 | 0.98 | 1.00[d] |
| | II | 1/2.5 | 5/2.3 | 4/5.1 | 10 | 1.17 | 0.87[d] |
| Trachea, bronchus and lung (162–163) | I | 64/54.7 | 42/43.5 | 70/77.8 | 176 | 0.77 | 0.13 |
| | II | 45/40.0 | 29/30.0 | 51/55.0 | 125 | 0.83 | 0.34 |
| Female reproductive organs (170–175) | I | 21/24.9 | 16/18.4 | 22/15.7 | 59 | 1.67 | 0.10 |
| Female breast (170) | I | 11/12.2 | 8/9.2 | 10/7.5 | 29 | 1.48 | 0.40 |
| Uterine cervix (171) | I | 5/3.7 | 2/2.1 | 1/2.2 | 8 | 0.29 | 0.28 |
| Uterine corpus (172) | I | 4/4.6 | 2/3.5 | 5/2.8 | 11 | 2.16 | 0.29 |
| Ovary (175) | I | 1/4.2 | 4/3.5 | 6/3.2 | 11 | 5.95 | 0.03 |
| Prostate (177) | I | 73/74.4 | 62/59.5 | 106/107.0 | 241 | 1.01 | 0.98[f] |
| | II | 59/59.1 | 48/48.3 | 89/88.6 | 196 | 1.02 | 0.97 |
| Kidney (180) | I | 13/13.4 | 6/10.3 | 23/18.2 | 42 | 1.43 | 0.35 |
| | II | 9/8.8 | 3/7.0 | 19/15.1 | 31 | 1.47 | 0.43 |
| Urine bladder (181) | I | 36/28.8 | 27/22.3 | 28/39.8 | 91 | 0.56 | 0.02 |
| | II | 22/21.9 | 20/15.5 | 22/26.6 | 64 | 0.81 | 0.48 |
| Melanoma (190) | I | 6/4.9 | 4/4.1 | 6/7.0 | 16 | 0.70 | 0.54 |
| | II | 3/2.7 | 4/3.5 | 4/4.8 | 11 | 0.73 | 0.66 |
| Other skin cancer (191) | I | 69/66.3 | 46/50.5 | 89/87.2 | 204 | 1.00 | 0.94 |
| | II | 51/46.5 | 31/35.0 | 66/66.5 | 148 | 0.91 | 0.62 |
| Nervous system including peripheral nerves (193) | I | 2/3.4 | 1/2.6 | 9/5.9 | 12 | 3.39 | 0.13 |
| | II | 2/2.3 | 1/1.9 | 5/3.9 | 8 | 1.71 | 0.56 |
| Thyroid (194) | I | 1/3.2 | 2/2.5 | 7/4.2 | 10 | 5.61 | 0.06 |
| | II | 1/1.9 | 1/1.4 | 4/2.7 | 6 | 3.71 | 0.26 |
| Lymphatic organs (200–202, 205) | I | 6/11.6 | 6/8.8 | 25/16.6 | 37 | 3.36 | 0.007 |
| | II | 6/ 9.3 | 4/6.8 | 19/12.9 | 29 | 2.77 | 0.04 |
| Multiple myeloma (203) | I | 6/7.2 | 5/5.8 | 12/10.0 | 23 | 1.47 | 0.44 |
| | II | 4/4.3 | 4/4.3 | 7/ 6.4 | 15 | 1.18 | 0.76 |
| Leukemia (204) | I | 12/10.0 | 7/8.6 | 14/14.5 | 33 | 0.81 | 0.60 |
| | II | 6/ 7.2 | 4/6.0 | 13/ 9.7 | 23 | 1.83 | 0.26 |

[a]I: Stratified on sex, age (10 year groups), and place of residence. II: Men only, stratified on age, residence, and cigarette smoking (never; ex-smoker; or 1–9, 10–19 or ≥20 cigarettes per day). [b]Estimated by logistic regression with three levels of milk consumption. [c]Two sided $P$ value for trend. [d]Departure from linear trend ($P<0.05$). [e]Rectosigmoid classified with colon (153). [f]Interaction with age (≥55 vs <55 years; $P<0.05$).

dietary variables as meat, egg, coffee or alcohol consumption.

Cancer of the prostate showed no association with milk consumption in analyses including all age groups. However, a significant interaction with age emerged ($P=0.03$), and for the 13 cases of cancer of the prostate diagnosed in men aged less than 55 years at the start of follow-up, our risk estimate indicated a strong positive association (OR = 14; $P=0.02$). For the remaining sites, associations did not differ significantly between sexes, and only results for males and females combined are shown in the tables.

A strong and significant positive association was observed between milk consumption and cancers of the lymphatic organs (OR = 3.4; $P<0.01$; 95% CI 1.4–8.2). The association was in particular found for lymphosarcomas, but not for reticulum cell sarcomas (Table IV). The few cases of Hodgkin's disease also displayed a strong association with milk intake. The relation between cancer of lymphatic organs and milk consumption was largely restricted to non-smokers, and it was stronger among subjects born in urban than in rural areas. Our data did not reveal any association between milk intake and leukaemia comparable to that with cancers of the lymphatic organs, despite an estimated positive relation after control for cigarette smoking (Table III). In particular, no excess risk of lymphatic leukaemia could be demonstrated among those with a high milk intake.

Cancer of the urinary bladder initially showed a significant inverse association with milk consumption (Table III). However, control for cigarette smoking weakened our estimate of this association (OR = 0.8; 95% CI 0.4–1.5). For kidney cancer, our general analysis suggested only a weak positive relation with milk intake (OR = 1.4; 95% CI 0.7–3.0).

A high and significant odds ratio estimate was found for cancer of the pharynx. No difference in risk was established between cases of cancer of the hypopharynx and other cases in this category. In addition, non-significant odds ratios (OR) exceeding 2.0 were seen for cancer of the lip, the nervous system and the thyroid. Cancer of the buccal cavity gave an odds ratio estimate less than 0.5.

**Table IV** Milk consumption and incidence of cancer in lymphatic organs[a]

| | Observed/expected no. of cases by milk consumption (glasses per day) | | | | | | |
|---|---|---|---|---|---|---|---|
| | <1 (0) | 1 (1) | ≥2 (2) | Total no. of cases | Odds ratio, ≥2 glasses per day vs <1 glass per day[b] | P for trend | P for homogeneity |
| All cases[c] | 6/11.2 | 6/8.6 | 24/16.2 | 36 | 3.2 | 0.01 | |
| Histological subtypes | | | | | | | 0.43[d] |
| Lymphosarcomas | 1/4.3 | 3/3.4 | 9/5.4 | 13 | 6.2 | 0.02 | |
| Reticulum cell sarcomas | 2/2.0 | 1/1.3 | 3/2.7 | 6 | 1.2 | 0.87 | |
| Hodgkin's disease | 1/1.6 | 0/1.3 | 5/3.0 | 6 | 4.5 | 0.21 | |
| Other and unspecified | 2/3.3 | 2/2.6 | 7/5.2 | 11 | 2.4 | 0.27 | |
| Age at start of follow-up | | | | | | | 0.24 |
| ≤64 years | 4/5.6 | 3/4.0 | 12/9.4 | 19 | 1.9 | 0.26 | |
| ≥65 years | 2/5.6 | 3/4.6 | 12/6.8 | 17 | 5.9 | 0.01 | |
| Place of birth | | | | | | | 0.24 |
| Urban | 1/2.6 | 1/1.9 | 5/2.5 | 7 | 6.4 | 0.07 | |
| Rural | 5/5.3 | 2/3.7 | 11/9.0 | 18 | 1.5 | 0.51 | |
| Sibship size | | | | | | | 0.01 |
| ≤6 | 0/4.9 | 2/4.0 | 14/7.2 | 16 | 35.9 | <0.001 | |
| ≥7 | 6/5.8 | 4/4.5 | 9/8.7 | 19 | 1.0 | 0.97 | |
| Cigarette smoking status | | | | | | | 0.08 |
| Never smoked | 0/3.8 | 2/2.9 | 11/6.3 | 13 | 22.0 | 0.004 | |
| Former smoker | 2/1.8 | 0/1.2 | 3/2.0 | 5 | 1.7 | 0.69 | |
| Current smoker | 4/3.5 | 2/2.5 | 4/4.0 | 10 | 0.8 | 0.85 | |

[a]Stratified on sex, age (10 year groups), and place of residence. [b]Estimated by logistic regression with three levels of milk consumption. [c]Except for one case classified as benign in one hospital. [d]For the three specific subtypes indicated.

Additional analyses carried out with a more detailed categorisation of milk consumption did not reveal further associations, except for colorectal cancer. In this case a weak inverse relation emerged after separation of the two categories corresponding to an intake of 2–3 and 4 or more glasses of milk per day (odds ratio 0.74 for 4 or more glasses versus less than 1). This relation was largely unaffected by control for meat and egg consumption (corresponding odds ratio 0.61).

For all major cancer sites, analyses restricted to histologically confirmed cases produced results similar to those in Table III. However, for lung cancer, with a marked reduction in the number of cases, the inverse association with milk consumption was strengthened (OR = 0.67 as compared with 0.84, with adjustment for smoking). This association was particularly strong for small cell carcinoma and squamous cell neoplasms. The associations with lung cancer in this material were studied more extensively by Kvåle *et al.* (1983).

### Discussion

No relation could be demonstrated between milk consumption and total cancer incidence in this study. Still, a few asscociations emerged with cancer at particular sites, both in positive and negative direction. Thus, lymphomas were associated with high milk consumption, whereas cancers of the female reproductive organs showed weak positive relations with milk intake. By contrast, bladder cancer and lung cancer were associated with low milk consumption.

The restricted number of cases among females makes it more difficult to reach firm conclusions for cancer in this sex. In general, similar positive associations with milk consumption were indicated for cancers of the breast, endometrium and ovaries. Ovarian cancer gave the strongest relation, which remained significant after control for other dietary variables. Case–control studies have previously shown inconsistent results for this cancer (Cramer *et al.*, 1984; La Vecchia *et al.*, 1987*b*; Mori *et al.*, 1988) For breast cancer, some case–control studies (Hislop *et al.*, 1986; Talamini *et al.*, 1984) but not all (Lubin *et al.*, 1981; La Vecchia *et al.*, 1987*a*) have shown positive associations with milk intake. The interaction between milk and alcohol consumption seen in one study (Lê *et al.*, 1986) was not supported by our data.

A significant positive relationship with milk consumption was observed for cancer of the prostate in the younger part of our cohort. A positive association was also found in a previous follow-up study (Snowdon *et al.*, 1984), whereas no clear association could be established in a case–control study (Schuman *et al.*, 1982).

For cancers of the lymphatic organs, results similar to those seen here were reported from a case–control study (Middleton *et al.*, 1986), where intake of vitamin A, calculated largely on the basis of milk and vegetable consumption, was associated with an increased risk of Hodgkin's disease and leukaemia. An excess risk of malignant lymphoma seen among agricultural workers in many countries has been related to the widespread occurrence of bovine leukaemia virus causing lymphosarcoma in cattle (Pearce *et al.*, 1987). The milk of naturally infected cows frequently contains such infectious virus, and human cells can be infected *in vitro* (Ferrer *et al.*, 1981). However, work based on DNA hybridisation techniques has failed to demonstrate any involvement of bovine leukaemia virus in lymphorecticular malignancies in children (Bender *et al.*, 1988).

Special analyses were carried out to assess possible interactions with the effect of milk consumption in the association with malignant lymphomas. Although no overall association could be demonstrated with the number of siblings for each individual, the association with milk consumption was essentially confined to subjects with a moderate number of siblings (Table IV). Thus any risk associated with a high milk intake may be related to a particular social environment during childhood, a hypothesis supported by the contrast seen between those born in urban and rural areas. This contrast completely dominated a similar but weaker difference between individuals living in urban and rural areas at the start of follow-up. In this connection it may be noteworthy that the risk of Hodgkin's disease seems to be higher in an environment which affords protection from infectious exposure in early childhood (Gutensohn & Cole, 1981).

Initial analyses of our data pointed to an inverse association between bladder cancer and milk consumption. In connection with a similar observation in a case–control study, Mettlin and Graham (1979) suggested that a protective effect of milk might be due to its vitamin A content. More recently, a significant inverse relation was found in one case–control study of bladder cancer (Slattery *et al.*, 1988), but not in another (Risch *et al.*, 1988). In our data the strength of the association was reduced by control for cigarette smoking, a major risk factor for bladder cancer. Further control for meat, egg or coffee consumption did not affect this relation.

The weak relationship between kidney cancer and milk intake was strengthened by exclusion of cancer of the renal pelvis (OR = 1.8 for ⩾2 glasses of milk per day versus less than one glass). This association was significant for the 25 cases in individuals aged less than 55 at start of follow-up (OR = 2.8; $P = 0.05$). Kidney cancer is inversely associated with coffee intake in our cohort (Jacobsen *et al*., 1986), but the relation indicated with milk consumption was not removed by control for coffee intake or cigarette smoking. A significant association between milk intake and cancer of the renal parenchyma was found in the case–control study of McCredie *et al*. (1988).

Our results are consistent with a weak inverse relation between colon cancer and milk consumption. It was only with an intake of ⩾4 glasses per day that milk seemed to have any noteworthy protective effect. It has been proposed that calcium ions from the diet may protect against toxic effects of fatty acids by forming insoluble compounds (Newmark *et al*., 1984). Several studies, including hospital-based case–control studies in Norway and Minnesota (Bjelke, 1973), have indicated inverse associations between colon cancer and milk consumption or overall calcium intake (Sorenson *et al*., 1988).

The dietary questionnaire used in this study did not elicit information on type of milk consumed. However, in the period before 1970, skimmed milk represented only a small fraction of the total amount of milk consumed in Norway, in contrast to a more widespread use earlier in this century (Statens Ernaeringsråd, 1985). Although minor changes may have occurred in milk consumption habits during follow-up, it is unlikely that such changes could have introduced any substantial bias in the associations considered. Norwegian whole milk contains approximately 3.8% fat, and in 1975 Norwegians received 14% of their dietary fat from milk (Solvang, 1984). The weak to moderate positive associations observed with cancers of the female reproductive organs and cancer of the renal parenchyma might be explained by the role of milk as a source of fat in Norway.

In this study, several dietary components known to be risk factors for certain types of cancer correlated strongly with milk consumption and thus were potential confounders. Yet adjustment for these factors, or for a factor such as occupation class, led to only minor changes in risk estimates for most cancer sites. Nonetheless, the conspicuous relations shown in Table II illustrate the potential of our data on milk intake for capturing essential associations with other variables, despite the fact that this information was collected once only, at the beginning of follow-up.

In our main statistical analyses, four out of 24 cancer sites showed a significant association with milk consumption at the 5% level. Routine computations of this kind, with a large number of tests, may easily lead to spurious associations. However, for most sites for which a significant relationship appeared, prior interest attaches to the potential association with milk intake or related variables. We have reported results for all major cancer sites in order to facilitate comparisons with other studies.

Our material included few individuals with a substantially increased milk consumption, and particular associations at the extreme part of the range for milk intake could remain undetected. At any rate, a total milk intake of the magnitude observed here cannot contribute very much to the overall risk in the population of developing cancer, although some negative effects could possibly be reduced by consuming milk with a lower fat content. The most striking result in our study is the association with cancers of the lymphatic organs. Although these are not very common cancers, the possibility that a specific aetiological agent could be transmitted to humans through cow milk warrants further research.

This work was supported by Public Health Service contract N01CP-91043 from the Division of Cancer Etiology, National Cancer Institute, by the Norwegian Cancer Society and by a visiting scientist award from the International Agency for Research on Cancer to E.B.

### References

BENDER, A.P., ROBISON, L.L., KASHMIRI, S.V.S. & 8 others (1988). No involvement of bovine leukemia virus in childhood acute lymphoblastic leukemia and non-Hodgkin's lymphoma. *Cancer Res*., **48**, 2919.

BJELKE, E. (1973). *Epidemiologic Studies of Cancer of the Stomach, Colon, and Rectum; with Special Emphasis on the Role of Diet. Vols 2–4*. University Microfilms: Ann Arbor, MI.

COMMITTEE ON DIET, NUTRITION AND CANCER. ASSEMBLY OF LIFE SCIENCES. NATIONAL RESEARCH COUNCIL. (1982). *Diet, Nutrition, and Cancer*. National Academy Press: Washington, DC.

CRAMER, D.W., WELCH, W.R., HUTCHISON, G.B., WILLETT, W. & SCULLY, R.E. (1984). Dietary animal fat in relation to ovarian cancer risk. *Obstet. Gynecol*., **63**, 833.

FERRER, J.F., KENYON, S.J. & GUPTA, P. (1981). Milk of dairy cows frequently contains a leukemogenic virus. *Science*, **213**, 1014.

GUTENSOHN, N. & COLE, P. (1981). Childhood social environment and Hodgkin's disease. *N. Engl. J. Med*., **304**, 135.

HISLOP, T.G., COLDMAN, A.J., ELWOOD, J.M., BRAUER, G. & KAN, L. (1986). Childhood and recent eating patterns and risk of breast cancer. *Cancer Detect. Prev*., **9**, 47.

JACOBSEN, B.K., BJELKE, E., KVÅLE, G. & HEUCH, I. (1986). Coffee drinking, mortality, and cancer incidence: results from a Norwegian prospective study. *J. Natl Cancer Inst*., **76**, 823.

KUMMET, T. & MEYSKENS, F.L. (1983). Vitamin A: a potential inhibitor of human cancer. *Semin. Oncol*., **10**, 281.

KVÅLE, G., BJELKE, E. & GART, J.J. (1983). Dietary habits and lung cancer risk. *Int. J. Cancer*, **31**, 397.

LA VECCHIA, C., DECARLI, A., FRANCESCHI, S., GENTILE, A., NEGRI, E. & PARAZZINI, F. (1987*a*). Dietary factors and the risk of breast cancer. *Nutr. Cancer*, **10**, 206.

LA VECCHIA, C., DECARLI, A., NEGRI, E. & 5 others (1987*b*). Dietary factors and the risk of epithelial ovarian cancer. *J. Natl Cancer Inst*., **79**, 663.

LÊ, M.G., MOULTON, L.H., HILL, C. & KRAMAR, A. (1986). Consumption of dairy produce and alcohol in a case–control study of breast cancer. *J. Natl Cancer Inst*., **77**, 633.

LUBIN, J.H., BURNS, P.E., BLOT, W.J., ZIEGLER, R.G., LEES, A.W. & FRAUMENI, J.F. (1981). Dietary factors and breast cancer risk. *Int. J. Cancer*, **28**, 685.

MCCREDIE, M., FORD, J.M. & STEWART, J.H. (1988). Risk factors for cancer of the renal parenchyma. *Int. J. Cancer*, **42**, 13.

MAGNUS, K., HOUGEN, A., ANDERSEN, A. & PEDERSEN, E. (1970). A study of disease in migrants and their siblings: development of sibling rosters. *J. Chronic Dis*., **23**, 405.

MANTEL, N. (1963). Chi-square tests with one degree of freedom: extentions of the Mantel–Haenzel procedure. *J. Am. Stat. Assoc*., **75**, 690.

METTLIN, C. & GRAHAM, S. (1979). Dietary risk factors in human bladder cancer. *Am. J. Epidemiol*., **110**, 255.

MIDDLETON, B., BYERS, T., MARSHALL, J. & GRAHAM, S. (1986). Dietary vitamin A and cancer–a multisite case–control study. *Nutr. Cancer*, **8**, 107.

MORI, M., HARABUCHI, I., MIYAKE, H., CASAGRANDE, J.T., HENDERSON, B.E. & ROSS, R.K. (1988). Reproductive, genetic, and dietary risk factors for ovarian cancer. *Am. J. Epidemiol*., **128**, 771.

NEWMARK, H.L., WARGOVICH, M.J. & BRUCE, W.R. (1984). Colon cancer and dietary fat, phosphate, and calcium: a hypothesis. *J. Natl Cancer Inst*., **72**, 1323.

PAMUKCU, A.M., ERTÜRK, E., YALCINER, S., MILLI, U. & BRYAN, G.T. (1978). Carcinogenic and mutagenic activities of milk from cows fed bracken fern *(Pteridium aquilinum)*. *Cancer Res*., **38**, 1556.

PEARCE, N.E., SHEPPARD, R.A., SMITH, A.H. & TEAGUE, C.A. (1987). Non-Hodgkin's lymphoma and farming: an expanded case–control study. *Int. J. Cancer*, **39**, 155.

RISCH, H.A., BURCH, J.D., MILLER, A.B., HILL, G.B., STEELE, R. & HOWE, G.R. (1988). Dietary factors and the incidence of cancer of the urinary bladder. *Am. J. Epidemiol*., **127**, 1179.

SCHUMAN, L.M., MANDEL, J.S., RADKE, A., SEAL, U. & HALBERG, F. (1982). Some selected features of the epidemiology of prostatic cancer: Minneapolis–St Paul, Minnesota case–control study, 1976–1979. In *Trends in Cancer Incidence*, Magnus, K. (ed) p. 345. Hemisphere Publishing: Washington, DC.

SLATTERY, M.L., WEST, D.W. & ROBISON, L.M. (1988). Fluid intake and bladder cancer in Utah. *Int. J. Cancer*, **42,** 17.

SNOWDON, D.A., PHILLIPS, R.L. & CHOI, W. (1984). Diet, obesity and risk of fatal prostate cancer. *Am. J. Epidemiol.*, **120,** 244.

SOLVANG, A. (1984). Diet and food expenditures for private households 1947–1981, an analysis based upon the Central Bureau of Statistics consumer reports of 1947–48, 1951–52, 1958, 1975 and 1981. Report no. 33. Institute of Nutritional Research: Oslo.

SORENSON, A.W., SLATTERY, M.L. & FORD, M.H. (1988). Calcium and colon cancer: a review. *Nutr. Cancer*, **11,** 135.

STATENS ERNAERINGSRAD (1985). *Mat i Norge*. NKS forlaget: Oslo (in Norwegian).

STOCKS, P. & KARN, M.N. (1933). A cooperative study of the habits, home life, dietary and family histories of 450 cancer patients and an equal number of control patients. *Ann. Eugenics*, **5,** 237.

TALAMINI, R., LA VECCHIA, C., DECARLI, A. & 5 others (1984). Social factors, diet and breast cancer in a northern Italian population. *Br. J. Cancer*, **49,** 723.

TARONE, R.E. (1975). Tests for trend in life table analysis. *Biometrika*, **62,** 679.

THOMAS, D.G. & GART, J.J. (1983). Stratified trend and homogeneity analyses of proportions and life table data. *Comput. Biomed. Res.*, **16,** 116.